# Identification of SARS-CoV-2 in urban rodents from Southern Mexico City at the beginning of the COVID-19 pandemic

**DOI:** 10.1590/S1678-9946202466008

**Published:** 2024-02-05

**Authors:** Fernando Martínez-Hernández, Nelly Raquel Gonzalez-Arenas, José Antonio Ocampo Cervantes, Guiehdani Villalobos, Angelica Olivo-Diaz, Emilio Rendon-Franco, Pablo Maravilla, Mirza Romero Valdovinos, Claudia Irais Muñoz-Garcia

**Affiliations:** 1 Hospital General “Dr. Manuel Gea González”, Ciudad de México, Mexico; 2 Universidad Autónoma Metropolitana, Centro de Investigaciones Biológicas y Acuícolas de Cuemanco, Ciudad de México, Mexico; 3 Universidad Autónoma Metropolitana, Departamento de Producción Agrícola y Animal, Ciudad de México, Mexico; 4 Hospital General “Dr. Manuel Gea González”, Departamento de Biología Molecular e Histocompatibilidad, Laboratorio de Patógenos Emergentes, Ciudad de México, Mexico

**Keywords:** SARS-CoV-2, Rattus norvegicus, Mus musculus, Synanthropic animals, Urban environments

## Abstract

Currently, there are some concerns about the situation and, in particular, about the future of the COVID-19 pandemic and the new emerging variants of SARS-CoV-2. Rodents are an example of synanthropic animals in urban environments that harbor important zoonoses. Although the molecular identification of SARS-CoV-2 in *Rattus norvegicus* from New York City had been reported, in other studies, urban wild rodents infected with this virus have not been found. This study aimed to molecularly identify the presence of SARS-CoV-2 in urban wild rodents from Mexico City, trapped along a water channel of a public park as part of a pest control program, at the beginning of the COVID-19 pandemic, during the fall and winter of 2020. Up to 33 *Mus musculus* and 52 *R. norvegicus* were captured and euthanized, large intestine samples with feces from the animals were obtained. RNAs were obtained and subjected to qRT-PCR for SARS-CoV-2 identification and threshold cycle (Ct) values were obtained. Four mice (12.1%) and three rats (5.8%) were positive, three rodents exhibited Ct<30. Our results on the frequency of SARS-CoV-2 in urban rats are in line with other previous reports. Thus, similar to other authors, we suggest that surveillance for the detection of SARS-CoV-2 in urban wild rodents, as sentinel animals, should be maintained.

## INTRODUCTION

On May 5th, 2023, the Director General of World Health Organization (WHO) announced that COVID-19 caused by the SARS-CoV-2 virus is no longer defined as a public health emergency of international concern. However, this virus still poses a significant threat to public health at a global level. Additionally, the emergence of new variants of the SARS-CoV-2 virus has caused further distress and concern among the global population^1^.

Even though SARS-CoV-2 primarily infects the respiratory tract in humans, the virus has also been identified in the gut tissue and feces of infected individuals. Moreover, the viral genome has also been detected in wastewater, which suggests potential transmission routes from human hosts to urban sewage fauna^2,3^. When human infections by pathogens infect animals, it is called reverse zoonoses or zooanthroponosis. If this phenomenon occurs in urban environments, complex dynamic interfaces are generated, which can promote the transmission of diseases between people and animals and vice versa^4^.

Synanthropic species thrive in urban environments and have adapted to the selection pressures imposed by such environments, often in response to available resources. However, these species can also carry zoonotic pathogens and, in some cases, act as reservoir hosts for these pathogens. Rodents, for instance, are a typical example of synanthropic animals that harbor significant zoonotic diseases^5^.

In addition, some rodent species, such as *Mesocricetus auratus*, *Mus musculus* (house mouse), *Myodes glareolus*, *Neotoma cinerea*, *Peromyscus maniculatus*, *P. leucopus*, and *Rattus norvegicus* (urban brown rat), have been experimentally infected with emerging variants of SARS-CoV-2^6^.

Although one research article reported finding molecular evidence of SARS-CoV-2 infection in urban brown rats^7^, other studies have shown inconsistent results. Some studies have found brown rats with positive serology but negative molecular detection for SARS-CoV-2^8,9^, in contrast to others which have not detected any positive rodents by either serology or molecular testing^10,11^. Interestingly, wild *M. musculus* have been reported to be resistant to ancestral strains of SARS-CoV-2, but laboratory experiments have revealed that these rodents are susceptible to the latest virus variants^12^. Therefore, this study aimed to identify the presence of SARS-CoV-2 in urban wild rodents in the south of Mexico City by a molecular approach at the beginning of the COVID-19 pandemic.

## MATERIALS AND METHODS

### Study area and sampling

This study was conducted during the fall and winter of 2020, as part of an ecological restoration program carried out along an open water channel *Canal Nacional* that has been transformed into a linear public park (approximately at 19°21’02” N, 99°07’11” W); the local authorities of Mexico City, together with the Universidad Autonoma Metropolitana (UAM), experts in native and invasive fauna, performed a rodent pest control program. This program was carried out under the 31112246 approval project within the agreement UAM-SAREVICH 322003.

The rodents were captured with commercial galvanized wire mesh box traps (Tomahawk Live Trap-like), measuring 30x20x14 cm, baited with oats, vanilla essence, peanut butter, and corn tortilla, anesthetized with chloroform and euthanized by cervical dislocation. The specimens were transferred to the laboratory for morphological identification, age classification (using weight and body length), and determination of reproductive status according to previously documented guides and reference values^13,14^.

Individual data were obtained and the dissection of some organs and the entire large intestine was carried out by surgically opening the bodies in a biosafety cabinet with laminar flow, and using personal protective equipment such as gloves, face masks, and lab coat; the samples were stored at –20 °C until use. For this study, only samples from the large intestine were processed (0.25–1g).

### RNA extraction and quantitative reverse transcription polymerase chain reaction (qRT-PCR) assays

RNA extraction was performed using an automated nucleic acid platform (Maelstrom 9600, TANBead, Taipei, TW) with an extraction commercial system (TANBead Viral Nucleic Acid Extraction Kit, Tiangen, Beijing, CN). To each rodent intestine sample, 300 μL of phosphate buffered saline (PBS) was added and mixed vigorously to obtain an intestinal lavage liquid, which was added to the wells of the kit’s Lysis Buffer (LB) plate, 10 μL of proteinase K (20 mg/mL) was added and the plate was kept at room temperature for 15 min. The extraction was continued as indicated in the manufacturing protocol; briefly, each of the plates contained in the extraction kit were placed in its corresponding position within the equipment and the extraction program was started, which consists of activation of the magnetic beads, lysis, washing, and elution of the RNA. The RNA obtained was placed in microtubes and stored at -70 °C until use.

The detection of SARS-CoV-2 in rodents was carried out by one-step qRT-PCR with the SARS-CoV-2 (Open Reading Frame, *ORF1ab*; and *Nucleocapsid*, *N* genes) real-time PCR detection Kit (Viasure, CerTest Biotec, Zaragoza, Spain), following the manufacturer’s instructions; briefly, the contents of the wells (specific primers/probes, dNTPS, buffer, polymerase, and reverse transcriptase) were solubilized with 15 μL of hydration buffer, and then 5 μL (300ng/μL) of RNA extracted from intestinal contents were added, 5μL of positive control and 5 μL of negative control, respectively (both controls contained in the kit). The amplification was carried out with the QIAquant real-time 96 thermal cycler (QIAGEN) with the following amplification program: 15 min at 45 °C for retrotranscription, 2 min at 95 °C for initial denaturation followed by 45 amplification cycles of 95 °C for 10 s and 60 °C for 50 s. Samples with a threshold cycle (Ct) of less than 40 for both genes were considered positive, as indicated by the manufacturer.

### Statistical analysis

The description of weight and sex is presented as the mean and standard deviation and ratios, respectively. SARS-CoV-2 frequencies are presented as percentages. To test differences in the frequency of sex, weight, and stage of development, two-tailed Fisher’s exact tests were performed. Data analysis was performed with Epi Info6 software tools (version 6.04, Centers for Disease Control and Prevention, USA).

## RESULTS

Samples from 33 *M. musculus* and 52 *R. norvegicus* were analyzed. Their mean weight and male/female ratio were 15.8±2.9g, 21/12 and 246.3±55.8g, 17/35 for *M. musculus* and *R. norvegicus*, respectively; seven mice and 14 rats were young animals, two rats and six mice were pregnant.

**Figure 1:**
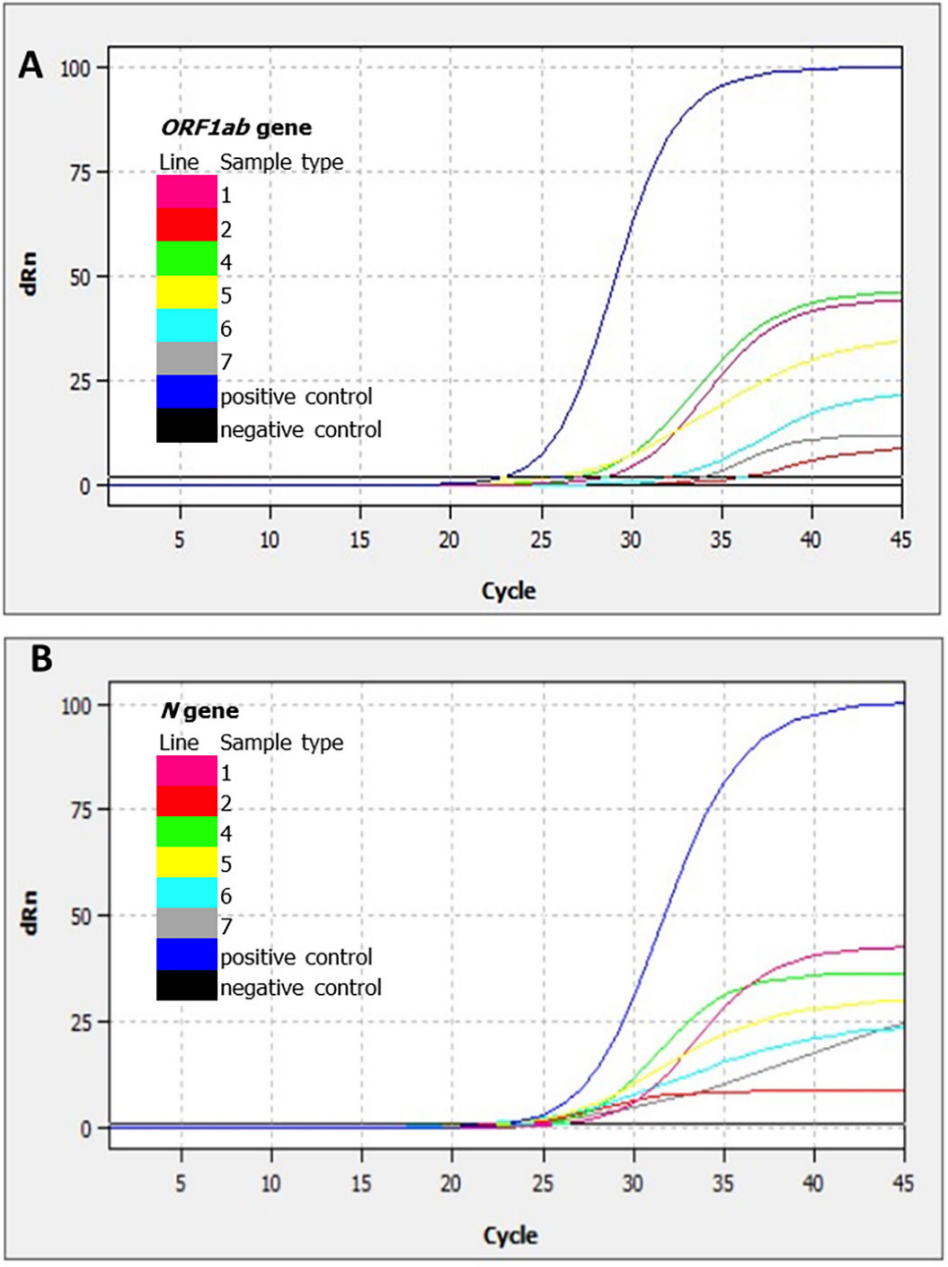
- RT-qPCR Amplification curve plots for SARS-Cov-2, showing fluorescent amplification measurement (dRn) vs PCR cycle numbers for different positive rodent samples (in colors). In A) *ORF1ab* gene amplification and B) *N* gene amplification are shown. Curves for sample 3 are not shown.

RNA from all samples was obtained and RT-qPCR for SARS-CoV-2 were performed. Seven samples were positive, i.e., 8.2% (7/85) for both species, divided into 12.1% (4/33) for mice and 5.8% (3/52) for rats; no positive rodent was pregnant. Table 1 summarizes the main findings, variables, and Ct values for both viral markers (*ORF1ab* and *N*); in three animals, both Ct values were lower than 30. Figure 1 shows the qRT-PCR for SARS-CoV-2 amplification plot for positive rodents. No variable (species, sex, weight, and stage of development) was statistically associated with SARS-CoV-2 infection (comparisons are not shown).

**Table 1:** - Rodents positive for SARS-CoV-2.

Nº	Species	Sex	Weight (g)	Stage of development	Ct	
					*ORF1ab*	*N*
1	*R. norvergicus*	Male	208	Adult	28	27
2	*R. norvergicus*	Female	268	Adult	36	27
3	*R. norvergicus*	Male	155	Young	36	24
4	*M. musculus*	Female	10	Adult	27	25
5	*M. musculus*	Female	17	Adult	26	23
6	*M. musculus*	Male	14	Adult	32	22
7	*M. musculus*	Male	19	Adult	34	23

## DISCUSSION

In the initial stages of the COVID-19 outbreak, there was a great deal of uncertainty regarding the zoonotic origin of the virus and the possibility of wild animals being infected and act as reservoirs for the SARS-CoV-2 virus^15,16^. Thus, based on the similarities of Angiotensin Converting Enzyme 2 (ACE2) and Transmembrane Protease, Serine 2 (TMPRSS2), both recognized as receptors and protease for the SARS-CoV-2 spike protein, wild rodents were proposed as having a high potential risk of infection by this virus^15^. However, empirical evidence obtained in a previous study^7^ and in this study suggests that urban wild rodents, such as the *M. musculus* and *R. norvergicus*, have a low rate of SARS-CoV-2 infection. Here, we found that 12.1% of *M. musculus* and 5.8% of *R. norvegicus* were infected with SARS-CoV-2. In a study performed with 79 *R. norvegicus* captured in New York City during the fall of 2021, showed that 16.5% had titers of IgG- or IgM-positive for SARS-CoV-2 and four rats (5%) were RT-qPCR positive^7^. Another study performed with 80 *R. norvegicus* and 69 mice (*Apodemus sylvaticus*) trapped in an urban park and sewage treatment facility in Liverpool, UK, showed that seven rats (8.7%) had positive titers of IgA in lung tissue fluid^8^, whereas in two of 213 (0.9%) thoracic cavity fluid samples of *R. norvegicus* from Windsor, Canada, neutralizing antibodies against SARS-CoV-2 were identified^9^.

When performing quantitative PCR, the Ct value is determined by the cycle number at which the fluorescent signal exceeds the background level. Generally, qRT-PCR protocols detect SARS-CoV-2 considering samples with Ct values of less than 40 positive for viral RNA. Despite the lack of a standard development curve regarding viral DNA concentration versus Ct in this study, it is recognized that Ct values may indirectly indicate the magnitude of the viral load in a given sample^17^. Many patients with severe COVID-19 infection treated at a general hospital in the Southern region of Mexico City in early 2020 presented Ct<30 values^18^. Interestingly, in this study, three rodents exhibited Ct<30 values. This result suggests that these rodents may have eliminated a substantial viral load in their feces. Notably, this hypothesis requires further experiments to be confirmed.

This work presents some limitations, i.e., it was not possible to obtain the rodents’ blood samples to perform serology and viral neutralization tests; on the other hand, the SARS-CoV-2 commercial molecular diagnostic kit used here was designed for use on human samples taken by nasopharyngeal swab (Certest Biotec, San Mateo de Gállego, Zaragoza, Spain), so it cannot be ruled out that the sensitivity is different from that reported by the supplier, since rodent fecal samples were tested. Also, sequencing analyses did not provided information about lineages or variants of SARS-CoV-2. In addition, the sample size of the rodents in this study was small and lacked representative sampling. Hence, caution is advised when interpreting the number of rodents that tested positive for SARS-CoV-2, as the prevalence may be higher in other rodent populations. In future studies, a larger sample size and a more diverse representation of rodents could provide more comprehensive insights into the prevalence of SARS-CoV-2 among wild rodents.

Currently, natural transmission of SARS-CoV-2 among animals has been described mainly in companion animals (e.g., cats and dogs) and in mink farms^19^. As the SARS-CoV-2 pandemic has evolved, several virus variants carrying multiple mutations have arisen worldwide; some variants have been classified by World Health Organization (WHO) as variants of interest (VOIs) or variants of concern (VOCs), based on epidemiological evidence of enhanced transmission and possible evasion of immunity, therefore, animal models play a key role in assessing VOC transmission, immune escape, and pathogenicity^1^. Although the first SARS-CoV-2 variants did not bind to mouse ACE2, the Omicron variant, emerging in late November 21presents the largest number of changes (45 point mutations) and some of them have been associated with mouse adaptation^20^; therefore, surveillance in wild animals is a pivotal process in the emergence of new viral variants^16^. However, there are many uncertainties regarding SARS-CoV-2 infection in wild rodents, such as the minimum viral load needed to cause disease in rodents or whether these synanthropic species act as viral reservoirs. It is of utmost importance to conduct further research, work collaboratively, and share our findings to better understand the virus and prevent future outbreaks to protect public health.

## CONCLUSION

Our findings from the research conducted in Mexico City at the onset of the COVID-19 pandemic revealed that *M. musculus* and *R. norvegicus* rodents had a relatively low rate of SARS-CoV-2 infection, with only 12.1% for mice and 5.8% for rats. Although detecting viral presence in the feces of rodents with Ct values similar to those found in human patients with COVID-19 is a concerning indication, it also presents an opportunity to use urban wild rodents as sentinel animals for continuity; this may allow for early detection of any potential emergence of new SARS-CoV-2 variants.
